# Quetiapine Addiction: A Case Report

**DOI:** 10.1192/j.eurpsy.2022.2136

**Published:** 2022-09-01

**Authors:** A. Chaara, M. Sabir, F. El Omari

**Affiliations:** 1 Arrazi Psychiatric Hospital, Addictology Department, Sale, Morocco; 2 University Mohammed V of Rabat Medicine school of Rabat Arrazi university psychiatric hospital of Salé CHU IBN SINA of Rabat, Addictology Department, RABAT, Morocco; 3 University Hospital Center Ibn Sina, Ar-razi Psychiatric Hospital, Salé, Morocco

**Keywords:** Addiction, quetiapine

## Abstract

**Introduction:**

Quetiapine has been the subject of case reports documenting its abuse. In Morocco, no study has been done showing the prevalence of this misuse. The methods of administration are diverse: oral or nasal, injection, inhalation, consumption with cannabis (smoked) or alcohol, combination with other drugs. The abuse is associated in 75% of cases with another product.

**Objectives:**

The objective of this work is to describe the management of quetiapine dependence, through a clinical vignette.

**Methods:**

Through a clinical vignette, and by reviewing the literature, we will describe the management of quetiapine addiction.

**Results:**

Treatment consists of reducing the consumption of this substance until stopping. When possible, it is recommended to change this antipsychotic to another with low abuse potential and low antihistamine properties such as haloperidol, risperidone or aripiprazole. If, however, this solution was inapplicable, then limit the quantity of tablets by prescribing smaller amounts of antipsychotics and increase the frequency of visits.

**Cope and relieve:**

Sometimes other medicines can be used to relieve potential withdrawal symptoms, including benzodiazepines or hypnotics to manage insomnia.

**Warnings :**

Ideally, the drug should be reduced gradually with a gradual and planned decrease in the dose taken over the months.

There should also be periodic evaluations.

**Long term treatment:**

Management must be biopsychosocial.

Treating comorbidities is a fundamental step in preventing relapse.

**Conclusions:**

It is a “prescription” use disorder! Each prescription should be carefully weighed and time bound. It seems important to be vigilant with regard to the dosages administered and the treatment regimens offered to the patients.

**Disclosure:**

No significant relationships.

